# A Case of Meningial Apoplexy; Post-Mortem Appearances

**Published:** 1860-12

**Authors:** Henry Lyster

**Affiliations:** Brooklyn, Michigan


### A CASE OF MENINGIAL APOPLEXY;

### Post-Mortem Appearances.

Reported by Dr. HENRY LYSTER, Brooklyn, Michigan.

At Springville, Lenewee Co., Mich., Oct. 23rd, at 4 P. M., 1860. Mr. F. Greenall, aged about forty years, a native of England, a robust looking farmer, six feet high, and weighing about one hundred and seventy pounds, was seen to fall immediately after having received one or two severe blows, from the fist of another strong man. The ground was apparently even in this place where the occurrence happened. After falling he was seen from a distance of two or three rods to move only once, and that was in the act of passing his hands over his face. For twenty minutes, the time he remained out upon the ground, he was noticed occasionally from a distance, but was not seen to move. He was then taken into a house by a couple of men, and placed on a chair. Finding that he could not sit up, they laid him on the floor, with a stick of wood, covered by a robe, under his head, and he remained in that position for twenty-four hours. During all this time he did not speak, and appeared to be entirely insensible. Occasionally various methods were adopted to rouse him during the day, but all without the least effect. The people in the house said they supposed that his condition was chiefly owing to having taken too much spiritous liquor; though most of the facts in the case were well known to them. The testimony before the coroner's jury, showed that only three or four glasses of beer had been taken by Mr. Greenall, and those just previous to the affray.

Twenty-four hours after the reception of the injuries, a physician was summoned. The pulse was found to beat forty per minute, the respiration was ten or twelve, pupils slightly contracted, complete anesthesia, with a decided perceptible difference between the temperature of the right and left side; the right being the coldest, though the legs were both cold. The head was not cold, and the cheeks puffed and collapsed, as the air was inspired or expired from the lungs. Stimulants were immediately resorted to externally, with effect; those given by the mouth, not being swallowed but choking the patient. The pulse in the course of three hours, arose to one hundred and twenty, when he was bled to the amount of lbj. from a large vein; the blood being very dark, and flowing slowly. In the course of a few minutes the pulse came down to eighty-five, and the body became warmer, and covered by a moist adherent perspiration; but heat could not be imparted to the right side from the neck to the foot. The pulse soon arose to one hundred and fifty, weak, soft and fluttering. The sphincter was relaxed while in this insensible state and fœces were passed. He continued in this condition, the respiration becoming more and more labored, until death; which occurred twelve hours after he was seen by the physician, and thirty-six hours after the injury.

The post-mortem examination was made twelve hours after death, by Dr. Sherwood, the attending physician, Dr. Crowell, and myself.

From the testimony given before the jury, a blow was supposed to have been given in the epigastic region, and another about the angle of the lower jaw. We therefore commenced the investigation by laying back the abdominal parieties. No traces of inflammation or other morbid changes were discovered; all the viscera appearing normal; the bladder filled; the intestines were somewhat distended with gas and very pale; the stomach containing a very little gas, not sufficient to inflate it; together with a few drops of a dark greenish looking fluid, which appeared to be blood acted upon by the gastric juice. The liver was healthy; and the gall bladder appeared full. Upon laying back the sternum, and cutting through the pericardium, two or three ounces of serous fluid escaped. The heart appeared normal, though the *columnæ carnæ* were more than usually developed, in both ventricles. The smooth pleural surface of the lungs seemed to have been entirely obliterated by a previous inflammation, in which adhesions were formed from the apex to the lower part of the lower lobes. So numerous and firm were they, that considerable difficulty was experienced in detaching them. The lobes were all united to each other at their edges; and though the intra-lobar surfaces preserved their characteristic nature, all gliding movement was entirely prevented. The lung tissue appeared healthy, with the exception of the lower portion of the lower lobes of both sides, which were congested, and only partially permeable to air, and were easily broken down.

We next proceed to the brain, which, from the pathology of the case, we expected would reveal the immediate cause of the fatal result; and it would have received earlier attention had it not been necessary from the evidence submitted, to make a thorough examination of the region of the stomach.

Upon removing the calvarium, extravasated blood coagulated into a firm dark clot, was found between the skull and the dura mater, anterior, superior, and lateral, to the anterior half of the right hemisphere of the brain. The extravasated blood had pressed, in almost an antero-posterior line, the dura mater, (viewing it superiorly), from the center of the right anterior fossa of the frontal bone, to the lambdoidal suture on the same side. On the base of the skull the dura mater was separated from its adhesions to the temporal bone, as far as the foramen spinosum. The clot of extravasated blood was now scraped off from the membrane, and was found to weigh *one pound eight and a half ounces* (avoirdupois). A stellated fracture, situated at about the middle of the squamous portion of the temporal bone, was now discovered. None of the bone was detached or depressed. From the point of fracture radiated six separate fractures: one extending to the foramen spinosum, two inches in length; probably ruptering the middle meningial artery, as it left the foramen. Another fracture, about one inch and one-half in length, down the curved sinus in the squamous portion of the temporal bone. Another, extending anteriorly toward the frontal, and then superiorly into the parietal bone, was about four and one half inches in length. Another, two and one-half inches in length, extended directly to the parietal bone. Two others, about one-third of an inch in length, extended the one posteriorly, and the other inferiorly. The longest fracture, pursuing a circuitous route from the lower anterior portion of the parietal bone, though the point of contact to the foramen spinosum was about six inches and one-half in length. The point of rupture in the blood vessels from the condition of the parts, was unfortunately not detected; but from the position of the extravasated blood upon the base of the skull, and from the fracture extending to the foramen spinosum, it was our opinion that the middle meningial artery had been ruptured. The weight of the extravasated blood was equal to half the average weight of the adult brain in the male; but in this instance the brain must have been several ounces above the average, as the skull seemed quite large. The right hemisphere must have been pressed and crowded into one-half its bulk, not estimating the lateral displacement caused by the pressure indirectly extending to the other hemisphere. The lateral ventricle of the compressed side was filled with serum, mingled with blood in a fluid state; that upon the left side was partially filled with the same fluid, less highly colored with blood. The other ventricles were not examined. The brain substance did not seem abnormal in appearance, or at all congested or infiltrated with blood.

In the case here as concisely described as I was able, considering the number of facts necessary to relate in order to give a clear and full impression of its character, the unusual amount of blood extravasated upon the dura mater, the great displacement of the anterior half of the right hemisphere, (which I think may be truly said, was pressed posteriorly and laterally into the rest of the brain substance;) and the great length of time, considering these two circumstances, after sustaining the injury till the fatal result, render it one possessing no little interest. From the history of the case, it would be supposed that there was at first great concussion of the brain before the extravasation had become sufficient to induce much pressure. Had this not been the case, would not the apoplectic symptoms have come on more slowly?

The chief point of interest in the case seems to be the amount of exhaustion. Sir Astley Cooper, in his lecture on Compression of the Brain, says: "In the specimen on the table before us, *three ounces, the largest quantity I have seen*, was effused under the dura mater." Dr. Abercrombie gives, as a large quantity, (in the cases which he has brought in, to illustrate his work upon Diseases of the Brain,) four and five ounces. M. Chevalier took three or four ounces from under the dura mater beneath the fentanelle, from a child eighteen months old. Abernethy relates a case in which eight or nine ounces were abstracted from beneath the dura mater in an adult. Among the few authors cited, these being the only ones that I have the privilege of consulting at the present time, I find no instance mentioned where the quantity extravasated exceeded nine ounces. These were, however, all *under* the dura mater; no exact quantity being given in those cases when it was *upon* the dura mater. The great displacement and necessary pressure caused by a foreign body, as it were, occupying nearly one-fourth of the cranial vault, would seem sufficient to cause a more suddenly fatal issue than in reality took place; although we admit that the direct pressure of the clot was not made upon any tract of brain substance giving origin to nerves necessary to vital action.

Two interesting queries have arisen in regard to this point, viz.: Was the apoplexy due to a concussion of the brain, until the pulse, (twenty-eight hours after the injury), began to raise; and was the amount of extravasation small until this period and then increased in another? Or was the blood extravasated to the amount discovered at the time of or immediately after sustaining the fracture?

